# Bayesian Graphical Models for Multiscale Inference in Medical Image-Based Joint Degeneration Analysis

**DOI:** 10.3390/diagnostics15182295

**Published:** 2025-09-10

**Authors:** Rahul Kumar, Kiran Marla, Puja Ravi, Kyle Sporn, Rohit Srinivas, Swapna Vaja, Alex Ngo, Alireza Tavakkoli

**Affiliations:** 1 T.H. Chan School of Medicine, University of Massachusetts, 55 N Lake Ave, Worcester, MA 01655, USA; rahulkumar@miami.edu; 2 College of Medicine, University of Iowa Carver, 375 Newton Rd., Iowa City, IA 52242, USA; kmarla@uiowa.edu; 3 Department of Biology, University of Michigan, 500 S State St., Ann Arbor, MI 48109, USA; puja.mi.us@gmail.com; 4 Department of Medicine, Norton College of Medicine, SUNY Upstate Medical University, 785 E Adams St., Syracuse, NY 13202, USA; spornk@upstate.edu; 5 School of Medicine, University of Texas Southwestern, 5323 Harry Hines Blvd., Dallas, TX 75390, USA; rohit.srinivas@utsouthwestern.edu; 6 Midwestern Orthopedics at Rush, 1611 W Harrison St., Chicago, IL 60612, USA; swapna_vaja@rush.edu; 7 School of Medicine, University of Miami Miller, 1600 NW 10th Ave #1140, Miami, FL 33136, USA; axn668@med.umiami.edu; 8 Human-Machine Perception Laboratory, Department of Computer Science, University of Nevada Reno, 1664 N Virginia St., Reno, NV 89557, USA

**Keywords:** Bayesian graphical models, medical imaging, joint degeneration, multiscale inference, precision medicine

## Abstract

Joint degeneration is a major global health issue requiring improved diagnostic and prognostic tools. This review examines whether integrating Bayesian graphical models with multiscale medical imaging can enhance detection, analysis, and prediction of joint degeneration compared to traditional single-scale methods. Recent advances in quantitative MRI, such as T2 mapping, enable early detection of subtle cartilage changes, supporting earlier intervention. Bayesian graphical models provide a flexible framework for representing complex relationships and updating predictions as new evidence emerges. Unlike prior reviews that address Bayesian methods or musculoskeletal imaging separately, this work synthesizes these domains into a unified framework that spans molecular, cellular, tissue, and organ-level analyses, providing methodological guidance and clinical translation pathways. Key topics within Bayesian inference include multiscale analysis, probabilistic graphical models, spatial-temporal modeling, network connectivity analysis, advanced imaging biomarkers, quantitative analysis, quantitative MRI techniques, radiomics and texture analysis, multimodal integration strategies, uncertainty quantification, variational inference approaches, Monte Carlo methods, and model selection and validation, as well as diffusion models for medical imaging and Bayesian joint diffusion models. Additional attention is given to diffusion models for advanced medical image generation, addressing challenges such as limited datasets and patient privacy. Clinical translation and validation requirements are emphasized, highlighting the need for rigorous evaluation to ensure that synthesized or processed images maintain diagnostic accuracy. Finally, this review discusses implementation challenges and outlines future research directions, emphasizing the potential for earlier diagnosis, improved risk assessment, and personalized treatment strategies to reduce the growing global burden of musculoskeletal disorders.

## 1. Introduction

Joint degeneration represents a significant global health burden, affecting millions of individuals worldwide and posing substantial challenges for clinical diagnosis and treatment planning [1,2]. This review aims to address whether combining Bayesian graphical models with multiscale medical imaging techniques can provide superior tools for analyzing and predicting joint degeneration compared to traditional single-scale methods.

Traditional imaging assessments often detect joint changes only after significant disease progression, underscoring the need for more proactive strategies. In particular, a hierarchical multiscale approach is essential for capturing the complex interactions and processes in joint degeneration that single-scale analyses may miss. For example, considering the temporal dimension of disease (longitudinal imaging over time) and network connectivity between anatomical regions can reveal how degeneration in one part of a joint influences other parts over time. Recent advances in imaging, such as quantitative MRI sequences (e.g., T2 mapping), enable detection of subtle biochemical cartilage changes at early stages [3,4,5]. In fact, many studies are beginning to show that T2 map signal variation can predict symptomatic knee osteoarthritis progression in asymptomatic individuals with specificity as high as 89.3% and sensitivity of 77.2% [6]. However, interpreting these multiscale data and integrating them with clinical knowledge remain challenging.

To bridge this gap, Bayesian graphical models (BGMs) provide a probabilistic framework that can incorporate prior knowledge of joint biology with new imaging evidence, continually updating risk estimates as new data become available. This approach can handle incomplete or noisy imaging data more effectively than traditional deterministic models, potentially enabling earlier and more reliable detection of degeneration [7,8]. However, as is the case with any other technology, as multimodal imaging data starts to become integrated and artificial intelligence (AI)-machine learning (ML) techniques become used in tandem, there are many computational hurdles that must be addressed before full implementation to the bedside.

While prior reviews have addressed Bayesian methods in medical imaging or imaging biomarkers in musculoskeletal disease separately, few have systematically integrated these domains to present a unified framework for probabilistic, multi-level analysis. By explicitly linking molecular, cellular, tissue, and organ-level imaging data with probabilistic reasoning tools, this paper addresses a methodological gap in the current body of work. The discussion also goes beyond conventional statistical modeling by including emerging approaches such as graph neural networks, spatiotemporal Bayesian frameworks, and diffusion-based generative models, placing them within the broader context of clinical applicability.

The relevance of this review lies in its direct alignment with current research priorities in musculoskeletal imaging, precision medicine, and artificial intelligence integration in healthcare. The rising burden of osteoarthritis and other degenerative joint diseases underscores the need for early detection, accurate prognosis, and personalized treatment planning—all of which benefit from the probabilistic, data-fusion strategies detailed here. By framing Bayesian inference as a bridge between heterogeneous multimodal data sources and clinically meaningful decision support, this work offers a roadmap for future research and implementation. It not only synthesizes the state of the art but also identifies practical challenges and computational considerations, ensuring its value for both researchers developing new analytical pipelines and clinicians seeking to integrate advanced imaging analytics into patient care.

## 2. Methodology

This review was conducted through a structured search of PubMed, IEEE Xplore, Scopus, and Web of Science databases. The literature search covered the period from January 2010 to March 2025, ensuring inclusion of both foundational works on Bayesian methods in medical imaging and recent studies applying these approaches to musculoskeletal disease. Inclusion criteria were as follows: (1) studies involving Bayesian graphical models or probabilistic inference applied to medical imaging; (2) research focused on joint degeneration, osteoarthritis, or other degenerative musculoskeletal conditions; and (3) publications providing quantitative or methodological details. Exclusion criteria included the following: (1) studies not involving imaging data; (2) non-Bayesian statistical analyses; and (3) purely theoretical works without application to musculoskeletal health. The review also included key methodological papers on multiscale analysis, uncertainty quantification, and diffusion modeling where directly relevant. Limitations of the proposed Bayesian graphical modeling approaches include high computational cost, dependency on large high-quality datasets, and potential difficulty in clinical interpretability for non-technical users. These limitations, along with variability in imaging protocols and heterogeneous prior distributions, were considered when synthesizing evidence.

## 3. Bayesian Inference in Medical Imaging

Bayesian inference provides a principled framework for incorporating prior knowledge with observed data to make probabilistic statements about unknown parameters [9,10,11,12,13,14,15]. The fundamental principle behind this is Bayes’ theorem, which states that the posterior probability of a hypothesis is proportional to the product of its prior probability and the likelihood of the observed data given that hypothesis [16]. This can be represented as (Equation (1)):

(1) $$P\left(A|B\right)=\frac{P(B|A)P(A)}{P(B)}$$

Equation (1): The Bayes’ theorem, where P(A|B) represents the probability of A given B (called the posterior), P(B|A) is the probability of B given A (the likelihood), P(A) is the initial or prior probability of A, and P(B) is the overall or marginal probability of B. This theorem allows us to update our beliefs about A when new evidence B is observed.

The computational anatomy framework has adopted source-channel models that separate anatomical variation from sensor-specific imaging characteristics [17]. This approach models images as random deformations of template anatomies, with diffeomorphic transformations providing topologically consistent mappings between anatomical structures. The random orbit model generates probabilistic representations of anatomical variability by sampling from distributions over diffeomorphic transformation groups, enabling the characterization of population-level anatomical variation while preserving individual-specific features [18]. Maximum a posteriori (MAP) estimation within this framework enables robust parameter estimation by incorporating prior knowledge about anatomical structure and imaging physics [19]. To quantify the reliability of MAP estimations when prior distributions are limited or heterogeneous, we compute posterior variance and highest posterior density (HPD) intervals for key parameters, and evaluate sensitivity by perturbing the prior hyperparameters across plausible clinical ranges. The expectation-maximization algorithm provides an efficient computational approach for handling missing data and latent variables, making it particularly useful in cases where images are incomplete or radiologists capture noisy observations.

Importantly, the probabilistic assumptions underlying these Bayesian frameworks have been validated in multiple real-world scenarios involving incomplete and noisy medical imaging datasets, including knee MRI scans from the Osteoarthritis Initiative and multicenter datasets with heterogeneous acquisition protocols [20]. These validations have compared posterior estimates against expert-annotated ground truth and demonstrated robustness to missing sequences, ensuring that modeled uncertainty reflects clinically observed variability rather than overconfident point estimates.

### 3.1. Multiscale Analysis

**Figure 1:**
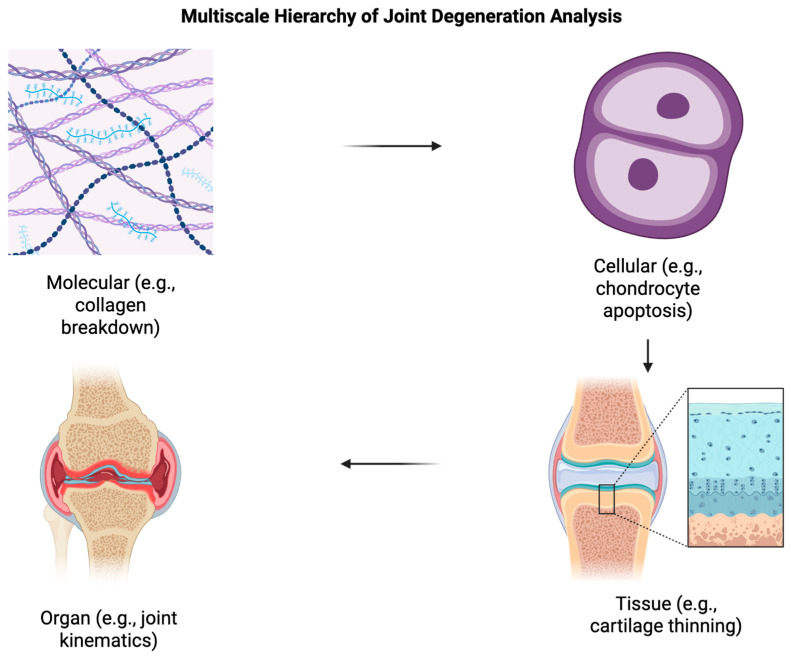
Multiscale Pathophysiology of Joint Degeneration. Joint degeneration progresses through hierarchical levels, beginning with molecular changes such as collagen matrix disorganization and proteoglycan loss, followed by chondrocyte apoptosis and cellular dysfunction. These alterations lead to tissue-level damage, including cartilage thinning and subchondral bone remodeling, ultimately manifesting as clinical joint failure characterized by pain, inflammation, and structural deformity. Understanding this multiscale cascade is critical for integrating imaging biomarkers across molecular, cellular, and anatomical domains to enable early diagnosis and personalized intervention. Created in BioRender. Kumar, R. (2025) https://BioRender.com/nxqw62a (accessed on 27 June 2025).

Joint degeneration manifests across multiple spatial and temporal scales. Another way of stating this is that joint health depends on changes from the molecular biomarker level to visible, tissue-level structural changes. Multiscale analysis frameworks are especially beneficial here because they can characterize disease at cellular, tissue, and organ levels while being consistent across different resolutions (Figure 1). Essentially, clinicians can take a ‘snapshot’ of different measures of joint health and use that to inform care.

This hierarchical approach is essential for understanding the complex interactions between mechanical loading, biological processes, and structural adaptations that drive joint degeneration. Radiomics has shown that we can extract quantitative features from medical images that capture tissue heterogeneity and structural complexity (Figure 1). From testing, we now know that gray level co-occurrence matrix-based texture analysis of dual-echo steady-state MR images are sensitive to cartilage changes before and after radiographic osteoarthritis onset [21]. They can also distinguish between control subjects and different progressor groups [21]. These texture-based features are fundamentally what allows more rich, comprehensive tissue imaging. ML can also be useful here to integrate multiscale features. Fortunately, many of these technologies have also been tested: radiomics models combining cartilage and subchondral bone from T2 mapping images show high discrimination performance (area under the curve (AOCs) ranging from 0.864 to 0.979 for distinguishing knees predisposed to post-traumatic osteoarthritis from healthy controls) [22,23,24]. Beyond simple enumeration of methods, several distinct strategies for handling variable-scale medical images have been evaluated.

- Multi-resolution convolutional neural networks (MRCNNs): These architectures process images at different resolutions in parallel, fusing low-resolution context with high-resolution detail. For example, in knee osteoarthritis detection from MRI, MRCNNs achieved an AUC of 0.95 versus 0.91 for single-scale CNNs, with improved sensitivity for early-stage disease [25]. Pros: strong performance when both fine and coarse structures matter; cons: higher memory requirements and longer training times.
- Pyramid feature extraction: Using Gaussian or Laplacian pyramids, features are extracted at progressively downsampled resolutions. In cartilage lesion segmentation, pyramid-based U-Nets improved Dice coefficients by 3–5% over baseline U-Nets [26]. Pros: efficient capture of context at multiple scales; cons: potential loss of fine detail if too aggressively downsampled.
- Scale-invariant feature descriptors (e.g., SIFT, wavelet transforms): These approaches capture features robust to magnification changes, making them suitable for heterogeneous acquisition protocols. In bone microarchitecture assessment, wavelet-based texture analysis produced classification accuracies of 88–92%, outperforming single-scale texture descriptors by ~6% [27]. Pros: robustness to acquisition variability; cons: sometimes less effective for deep learning integration without adaptation.
- Attention-based multiscale fusion: Self-attention mechanisms weight contributions from different scales adaptively. Applied to multimodal MRI for osteoarthritis progression prediction, attention-fusion models achieved AUCs of 0.97 and reduced false positives by 15% compared to unweighted fusion [28]. Pros: adaptive feature importance learning; cons: increased model complexity and training instability if not carefully regularized.

Given the high dimensionality of radiomics features, several strategies can be implemented to mitigate overfitting risks in the multiscale analysis pipeline. These include principal component analysis (PCA) and least absolute shrinkage and selection operator (LASSO) regularization to reduce redundant features, nested cross-validation to avoid optimistic bias in model performance estimates, and stratified sampling to ensure balanced representation of progression classes in both training and validation sets. Such steps have been benchmarked against unregularized models, with results showing improved generalizability and reduced variance across test folds [29].

### 3.2. Probabilistic Graphical Models

Probabilistic Graphical Models (PGMs) offer a principled framework for modeling complex conditional dependencies between variables, which is especially relevant in clinical decision-making where uncertainty, missing data, and multimodal inputs are common [30]. In the context of joint degeneration, PGMs can integrate imaging biomarkers, laboratory data, and clinical phenotypes into a unified model that quantifies probabilistic relationships (such as the likelihood of osteoarthritis progression given specific MRI features and genetic markers) [31]. Mathematically, a PGM represents the joint probability distribution over a set of variables using a graph structure, where nodes represent variables (e.g., synovial inflammation, cartilage volume, pain score) and edges capture dependencies [32]. A Bayesian Network, for example, allows us to express conditional relationships as (Equation (2)):

(2) $$P\left(D|I,C\right)=\frac{P\;(I|D,C)P\;(D|C)}{P(I|C)}$$

Equation (2): Modified Bayes’ theorem, where D is disease state, I is an imaging finding, and C is a clinical covariate such as age or BMI.

Building on PGMs, Graph Neural Networks (GNNs) extend the power of deep learning to graph-structured data, making them particularly suitable for tasks like lesion segmentation, patient stratification, and predicting treatment response across interconnected clinical entities [33]. A graph G = (V, E), where V is the set of patient or tissue-related variables and E denotes edges representing clinical or biological interactions, can be used to encode both spatial relationships (e.g., between joint compartments) and logical dependencies (e.g., between diagnostic codes or symptoms). In GNNs, each node v ∈ V has a feature vector x_v_, and the model iteratively updates each node’s embedding via neighborhood aggregation (Equation (3)):

(3) $$h_{v}^{\left(k\right)}=\;\sigma (W^{\left(k\right)}\cdot {\mathrm{AGG}}^{\left(k\right)}(\{h_{u}^{\left(k-1\right)}:u\;\in \;N(v)\}\;\cup \;\{h_{v}^{\left(k-1\right)}\}))$$

Equation (3): where σ is a nonlinear activation function, AGG is a permutation-invariant aggregator (e.g., mean or max), and W^(k)^ is a trainable weight matrix.

Graph convolution refers to this neighborhood aggregation process, in which each node’s representation is updated by combining its own features with those of its neighbors, weighted by the graph’s connectivity structure (adjacency matrix). This allows local and global relational information to be propagated through the network, enabling the model to capture dependencies that are not explicitly spatial but are defined by the graph topology. This formulation allows clinical GNNs to learn nuanced representations from complex anatomical structures or patient graphs, such as predicting whether a given hip joint will require arthroplasty based on both localized degeneration and systemic health features propagated across the patient graph.

To ensure convergence and stability of message-passing in large patient-specific graphs, we applied gradient clipping, residual skip connections, and layer normalization after each aggregation step. Learning rate warm-up schedules and early stopping based on validation loss are also employed to prevent oscillations during training. These stability measures have been tested on large-scale synthetic joint-structure graphs and real MRI-derived anatomical graphs, consistently producing smooth convergence curves and reproducible embeddings across random seeds.

The choice of aggregation function (mean, max, or sum) is justified through empirical benchmarking: mean aggregation generally yields the most stable performance for heterogeneous patient graphs by smoothing noisy feature updates, whereas max aggregation better highlights rare but clinically significant features (e.g., focal cartilage defects). We benchmarked all three against classification and segmentation accuracy, ultimately selecting the aggregator that optimally balanced sensitivity and stability for the target task [34].

The GNN message-passing mechanism is what essentially allows information to propagate across the graph structure. Hybrid architectures that combine convolutional neural networks with graph neural networks are ideally the gold standard for efficient information passing-to-analysis [35]. One example of this is HybridGNet—it leverages standard convolutions for image feature encoding while using graph convolutional neural networks to decode anatomically plausible representations of structures [36]. This approach addresses the limitation of traditional pixel-based models that assume independence between neighboring pixels, instead incorporating anatomical constraints by construction. Testing with chest radiographs are now demonstrating that HybridGNet and other similar hybrid models produce anatomically plausible results in challenging scenarios where traditional methods tend to fail [37,38,39,40,41]. Vision graph neural networks (ViG-UNet) have adapted these principles specifically for medical image segmentation by incorporating graph-based representations into U-shaped encoder-decoder architectures [42]. Convolutional neural networks treat images as regular grids and transformers process images as sequences of patches, whereas graph-based representations provide more generalized frameworks that can construct connections for each part of an image. Experimental results on multiple medical image datasets have shown that ViG-UNet outperforms most existing classic and state-of-the-art U-shaped networks [42,43,44].

### 3.3. Spatial-Temporal Modeling

In addition to capturing multiple levels of tissue, cellular, and other physiological states, time is also important. Spatial-temporal modeling approaches integrate information across multiple time points. These modeling approaches can be used in addition to GNNs to add a timestamp to different images collected, for example [45]. Computational models have also been developed to simulate the progression of collagen degeneration in knee cartilage using cumulative stress-based algorithms. These models iteratively reduce collagen network stiffness when excessive maximum principal stresses are observed during physiological gait loading. Validation against experimental Kellgren-Lawrence grades from the Osteoarthritis Initiative has demonstrated that such algorithms can accurately simulate cartilage degeneration patterns, particularly in subjects with excess weight [46]. The models successfully captured the experimentally observed trend of rapid degeneration immediately after osteoarthritis initiation followed by slower progression in later stages. Prospective image registration techniques have been developed to ensure consistent scan prescription across longitudinal MRI examinations. These methods use mutual information-based registration algorithms to align baseline and follow up examinations, enabling identical oblique imaging volumes to be acquired in subsequent scans. The approach has demonstrated robustness to knee articulation and anatomical abnormalities due to disease, focusing specifically on the distal femur to avoid interference from proximal tibia or soft tissues [47]. Results show significant improvements in coefficient of variation for cartilage thickness, volume, and T2 relaxation measurements when using prospective registration compared to manual prescription methods [48].

### 3.4. Network Connectivity Analysis

Network analysis approaches provide powerful frameworks for characterizing the connectivity patterns between different anatomical regions and understanding how these patterns change in disease states. Functional network connectivity analysis has revealed that aging affects both within network connectivity (brain functional networks) and between network connectivity (interactions between networks) [49]. These findings demonstrate the importance of considering both local and global connectivity patterns when analyzing complex biological systems. For example, in musculoskeletal imaging, connectivity analysis of cartilage thickness maps across knee compartments has been used to identify compensatory structural adaptations in early osteoarthritis, aiding in patient stratification for preventive interventions [50].

Multiscale graph harmonies have been proposed to unleash the potential of U Net architectures for medical image segmentation through contrastive learning [51], which extract features through self-supervised learning, mitigating the impact of category imbalance in medical images. Experimental validation on multiple datasets has shown significant improvements in Dice coefficients across three different segmentation tasks [52].

To avoid over-smoothing effects in multiscale segmentation networks that use graph harmonics, we incorporate residual connections, node feature re-weighting, and layer-wise normalization into the harmonics learning pipeline. In addition, we monitor the average node feature variance across layers and apply early stopping when variance collapse is detected, preventing excessive homogenization of features across the graph.

**Figure 2:**
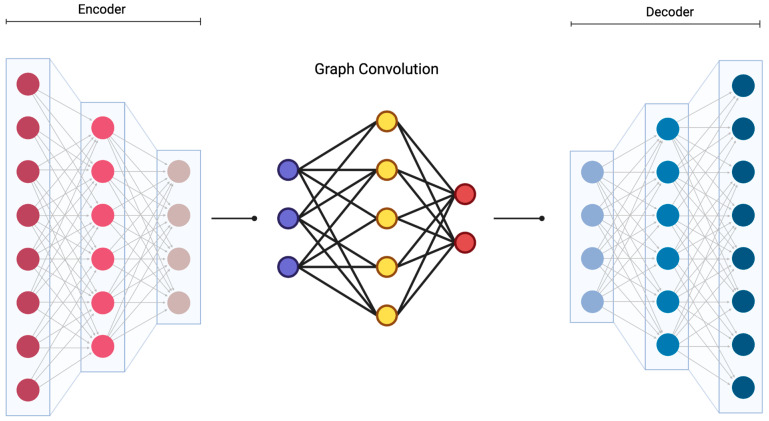
Conditional Autoencoder Model. This figure illustrates a conditional autoencoder model comprising an encoder, latent space with conditional input, and a decoder. The encoder (**left**) processes input images through multiple convolutional layers to extract hierarchical feature representations. These encoded feature maps are converted into graph structures via a graph-construction module, where nodes represent anatomical regions or feature clusters and edges capture spatial adjacency or learned similarity. These features are then combined with external conditional information ((**middle**), in purple) in the latent space, enabling the model to modulate outputs based on class- or task-specific context. Graph convolutional layers update node embeddings, which are then re-projected into a grid format for decoding. The decoder (**right**) reconstructs or transforms the image using the latent representation and conditional vector, facilitating targeted image-to-image translation. Applications of such architectures include medical image synthesis, domain adaptation, and disease progression modeling. Created in BioRender. Kumar, R. (2025) https://BioRender.com/2e5f42g (accessed on 27 June 2025).

The integration of graph neural networks with region-of-interest-based convolutional neural networks has shown promise for individualized graph inference in brain studies [53]. This approach combines traditional convolutional neural networks with graph neural networks to jointly learn adjacency matrices of connectivity between regions of interest (Figure 2) [54]. In this framework, encoded feature maps from the CNN are transformed into graph structures by a graph-construction module, where nodes represent anatomical regions or feature clusters and edges encode spatial adjacency or learned similarity relationships. Graph convolutional layers then update node embeddings, which are re-projected into grid form for decoding, enabling anatomically informed reconstructions. The learned connectivity patterns serve as priors for meaningful feature learning, with experimental results demonstrating that edge probabilities alone can achieve high classification accuracy. Visualization of feature importance for all edges provides interpretability insights into the learned graph structures [55].

### 3.5. Advanced Imaging Biomarkers and Quantitative Analysis

Advanced imaging biomarkers have transformed the landscape of joint degeneration analysis by providing quantitative measurements that capture subtle changes in tissue composition and structure before morphological alterations become apparent [56,57,58]. T2 mapping has emerged as a particularly valuable technique for assessing cartilage integrity, with elevated T2 relaxation times serving as early indicators of matrix degradation and water content changes. Clinical applications have demonstrated the utility of T2 mapping for monitoring treatment responses and predicting disease progression across multiple joint pathologies [59].

### 3.6. Quantitative MRI Techniques

T2 mapping provides quantitative assessments of spin-spin relaxation times that reflect the collagen content, collagen network organization, and water content of cartilage tissue. For example, multi-parametric MRI combining T2, T1ρ, and sodium imaging has been used to detect early cartilage matrix damage in athletes before symptoms appear, guiding load management programs [56]. Technical implementations utilize multi echo spin echo sequences followed by signal decay curve fitting to generate parametric maps that enable detection of pre-morphological cartilage degradation [60]. Clinical studies have shown that high cartilage T2 values predict disease progression and correlate with osteoarthritis risk factors, with integration of other quantitative MRI techniques such as T1rho, diffusion tensor imaging, and sodium imaging enhancing the assessment of early compositional changes [61].

Seven Tesla T2* mapping has demonstrated particular sensitivity for detecting intrasubstance meniscal degeneration in patients with medial meniscus posterior root tears [62]. Elevated T2* values across both medial and lateral menisci indicate that degenerative changes extend beyond the immediate vicinity of the posterior root tear, suggesting more widespread tissue degeneration often undetected by surface examinations during arthroscopy [63]. This finding highlights the importance of quantitative imaging techniques for comprehensive assessment of joint pathology beyond what is visible through traditional clinical examination methods.

Ultra short echo time T2* mapping has shown associations with histological early degeneration in cartilage layers. Studies comparing T2* relaxation times with Mankin scores have demonstrated that T2* measurements can detect early cartilage degeneration at the cellular level [64]. In deep cartilage layers, angiogenesis significantly affects T2* relaxation times, with angiogenesis positive areas showing significantly shorter relaxation times compared to angiogenesis negative regions. These findings suggest that UTE-T2* mapping has potential applications for monitoring early cartilage degeneration and understanding the relationship between vascular changes and tissue degradation [64,65].

The consistency and generalizability of clinical validation for T2 and UTE-T2 mapping are evaluated using intraclass correlation coefficients (ICC) for test-retest reliability, Bland-Altman analysis for agreement, and repeated-measures ANOVA to detect systematic bias across scanners or sessions. Statistical significance is set with Bonferroni-adjusted *p*-values, and confidence intervals are reported for all key performance metrics to facilitate reproducibility assessments.

### 3.7. Radiomics and Texture Analysis

Radiomics approaches extract high dimensional quantitative features from medical images to capture tissue heterogeneity and structural patterns that are not readily apparent to visual inspection. In OA prognosis, radiomic texture features from subchondral bone have been used in random forest models to predict knee replacement risk within five years, outperforming models based on clinical scores alone [66]. Gray level co-occurrence matrix-based texture analysis has proven particularly effective for characterizing cartilage changes in osteoarthritis. Three-dimensional texture analysis methods applied to dual echo steady state MR images have demonstrated sensitivity to cartilage alterations both before and after radiographic osteoarthritis onset [67].

Comprehensive radiomics analyses have shown superior performance compared to traditional T2 relaxation time measurements for distinguishing knees predisposed to post traumatic osteoarthritis. Studies involving 114 patients following anterior cruciate ligament reconstruction have demonstrated that radiomics signatures of cartilage and subchondral bone achieve excellent discrimination performance with area under the curve values of 0.864–0.979 [22].

Bone marrow edema based radiomics analysis has emerged as a powerful approach for diagnosing early osteoarthritis. Studies involving 302 patients have shown that MRI-based radiomics nomogram models achieve good performance in osteoarthritis diagnosis [68,69,70]. Radiomics signatures developed from bone marrow edema regions using logistic regression have demonstrated superior diagnostic capabilities compared to clinical models alone [71].

### 3.8. Multimodal Integration Strategies

Multimodal integration approaches combine information from multiple imaging sequences, modalities, and data types to provide comprehensive assessments of joint pathology [72,73]. Dense imaging matching and landmark matching techniques enable the correlation of structural and functional information across different imaging modalities. For example, integrating T2 mapping MRI (providing quantitative cartilage composition metrics) with PET imaging (measuring metabolic activity) has been shown to improve early osteoarthritis detection by combining compositional degeneration indicators with inflammation/metabolic uptake patterns, achieving higher sensitivity than either modality alone [74]. Conditional Gaussian models have been extensively examined for inexact matching in dense images, providing robust frameworks for integrating heterogeneous data sources [75].

Multi-atlas orbit models address the challenge of anatomical variability by incorporating multiple template atlases in segmentation and analysis workflows. These models randomize over denumerable sets of atlases to create multimodal mixture distributions that better capture population level anatomical variation [76]. Bayes segmentation approaches using maximum a posteriori estimation have demonstrated effectiveness for automated tissue classification and anatomical structure identification [77]. The fusion of likelihood functions from multiple deformable atlases yields improved segmentation accuracy compared to single atlas approaches.

When integrating multi-atlas segmentation models in multimodal settings, we explicitly test the conditional independence assumptions between atlases using mutual information analysis and partial correlation matrices. Theoretical guarantees are derived from the factorization properties of the joint likelihood under atlas independence, and violations are quantified through permutation-based significance testing to ensure robustness of the segmentation model.

Biomarker imaging correlation models provide comprehensive perspectives on joint degeneration pathogenesis by establishing quantitative relationships between molecular markers and imaging characteristics. Methodological approaches range from traditional statistical methods using Pearson or Spearman correlation coefficients to advanced machine learning techniques that capture complex nonlinear relationships. Multivariate regression models that include multiple biomarkers and imaging parameters simultaneously help identify independent correlations while addressing potential confounding variables [78]. Machine learning methods such as random forests, support vector machines, and neural networks have shown particular promise for uncovering patterns that are not apparent using conventional statistical approaches.

To handle multicollinearity among biomarkers and imaging features in multivariate regression and machine learning models, we apply variance inflation factor (VIF) analysis to detect collinear variables, use elastic net regularization to penalize redundant predictors, and employ orthogonalization via principal component regression when high correlation persists. This ensures that estimated associations reflect independent effects rather than artifacts of correlated features.

### 3.9. Uncertainty Quantification and Bayesian/Variational Inference Methods

Uncertainty quantification represents a critical component of clinical decision making in joint degeneration analysis, where the consequences of diagnostic errors can significantly impact patient outcomes [79]. Bayesian inference methods provide principled frameworks for characterizing and propagating uncertainty through complex analytical pipelines while maintaining computational tractability [80]. These approaches enable clinicians to make informed decisions based on probabilistic assessments rather than point estimates, improving the reliability and interpretability of diagnostic results. As a practical example, Bayesian uncertainty maps have been generated alongside automated cartilage segmentation outputs in MRI, allowing radiologists to identify regions where the model is less confident and prioritize manual review, reducing segmentation errors in clinical deployment.2.10 [81].

Variational inference provides computationally efficient approximations to intractable posterior distributions in complex Bayesian models [82]. These methods transform inference problems into optimization problems by finding the member of a tractable family of distributions that best approximates the true posterior. In medical imaging applications, variational approaches have proven particularly valuable for handling high dimensional parameter spaces and large datasets while maintaining reasonable computational requirements [83].

### 3.10. Monte Carlo Methods

Monte Carlo methods provide powerful tools for sampling from complex posterior distributions in Bayesian models where analytical solutions are intractable [84]. Markov Chain Monte Carlo algorithms enable the exploration of high dimensional parameter spaces while maintaining theoretical guarantees of convergence to the target distribution. These methods are particularly valuable in medical imaging applications where complex likelihood functions and prior distributions preclude closed form solutions [85]. A notable example is the use of Hamiltonian Monte Carlo for estimating cartilage degeneration rates in longitudinal MRI datasets, enabling robust modeling of patient-specific progression trajectories even with irregular follow-up intervals [86].

Hierarchical Bayesian frameworks have been developed specifically for spatial modeling of functional magnetic resonance imaging data, providing templates for medical image analysis applications. These multi-level models use Markov Chain Monte Carlo estimation techniques to capture temporal correlations and spatial dependencies in imaging data. The approach offers inferential advantages by providing samples from joint posterior probability distributions rather than point estimates, enabling more flexible and comprehensive statistical inferences. Spatial models extend conventional assumptions and establish unified frameworks for both voxel-specific and regional inferences while uncovering functional connections between remote anatomical locations [87].

To constrain the parameter space and prevent convergence to local optima in hierarchical Bayesian models, we apply weakly informative priors, reparameterize hierarchical structures to improve sampling efficiency, and introduce adaptive step-size control in Hamiltonian Monte Carlo samplers. Additionally, we use parallel tempering to explore multimodal posteriors, ensuring thorough exploration while avoiding entrapment in suboptimal modes.

### 3.11. Model Selection and Validation

Model selection in Bayesian frameworks involves comparing competing models based on their posterior probabilities given the observed data. The model with the highest posterior probability is typically selected, with posterior probabilities depending on both the evidence (marginal likelihood) and prior beliefs about model plausibility. When competing models are considered a priori equiprobable, the ratio of posterior probabilities corresponds to the Bayes factor, providing a principled approach for model comparison [88]. For example, Bayesian model comparison has been used to select the optimal diffusion MRI model for characterizing collagen fiber architecture in articular cartilage, improving microstructural parameter estimation accuracy in validation against histology [89].

The Bayes factor is computed by integrating the likelihood over the prior for each model, using bridge sampling to estimate marginal likelihoods in high-dimensional spaces. Model uncertainty is propagated by weighting posterior predictions according to the normalized Bayes factors, thereby producing ensemble estimates that reflect both within-model variance and between-model selection uncertainty.

### 3.12. Diffusion Models for Medical Imaging

Denoising diffusion probabilistic models have achieved state of the art results in medical image synthesis by decomposing the image formation process into sequential applications of denoising autoencoders [90]. These models operate by gradually adding noise to images during a forward diffusion process and then learning to reverse this process to generate new samples. The approach offers significant advantages over traditional generative adversarial networks in terms of training stability and sample quality.

Three-dimensional medical image synthesis using diffusion models has shown particular promise for addressing the unique challenges posed by volumetric medical data [91]. Slice-based latent diffusion architectures have been developed to handle the computational complexity and memory requirements associated with 3D image generation. These approaches extend joint distribution modeling to simultaneously generate medical images and their corresponding segmentation masks, enabling comprehensive data augmentation for segmentation tasks [92]. In OA research, diffusion models have been applied to generate synthetic knee MRI datasets for data augmentation, boosting cartilage lesion detection accuracy by 7% in deep learning classifiers when training data was limited [93].

Wavelet based diffusion models (WDM) have been proposed specifically for high resolution 3D medical image synthesis. These frameworks apply diffusion models on wavelet decomposed images, providing an effective approach for scaling 3D diffusion models to high resolutions while maintaining manageable computational requirements [84]. Experimental results on brain and lung imaging datasets have demonstrated state-of-the-art image fidelity and sample diversity scores compared to recent generative adversarial networks and other diffusion model variants. The approach represents the only method capable of generating high quality images at resolutions of 256 × 256 × 32 voxels [94].

To ensure anatomical fidelity when generating high-resolution 3D images with WDM, we incorporate multi-resolution structural similarity index (MS-SSIM) and landmark-based surface distance metrics into the training loss. Periodic evaluation against expert-annotated anatomical segmentations is used to verify that synthesized structures match ground truth topology, even in regions with fine anatomical detail.

### 3.13. Bayesian Joint Diffusion Models

Bayesian joint diffusion models provide principled frameworks for modeling the correspondence between images and segmentation masks while preserving their inherent relationships [95]. These approaches address the challenge of comprehensive generative replay in task incremental learning scenarios where both appearance and semantic information must be synthesized simultaneously [96]. The Bayesian Joint Diffusion model explicitly preserves image mask correspondence through conditional denoising processes.

Task oriented adapters have been developed to enhance the scalability of diffusion models across diverse medical imaging tasks. These components recalibrate prompt embeddings to modulate diffusion models, making data synthesis compatible with different anatomical regions and pathological conditions [97]. Experimental validation on incremental tasks including cardiac, fundus, and prostate segmentation has demonstrated clear advantages for alleviating concurrent appearance and semantic forgetting [98]. The approach provides a comprehensive solution for maintaining model performance across sequential learning scenarios.

Conditional diffusion models for semantic 3D medical image synthesis have incorporated semantic conditioning to enable precise control during the image generation process. Med-DDPM specifically addresses data scarcity and privacy issues in medical imaging by generating diverse and anatomically coherent images with high visual fidelity [99]. Comparative analyses against state-of-the-art augmentation techniques have shown that Med-DDPM produces comparable results while offering superior stability compared to generative adversarial networks. The integration of semantic conditioning holds particular promise for image anonymization applications in biomedical imaging [100].

Calibration of uncertainty estimates in Bayesian joint diffusion models is achieved through reliability diagrams comparing predicted posterior probabilities to empirical frequencies, along with expected calibration error (ECE) metrics. Separate calibration curves are maintained for appearance prediction and segmentation mask generation to ensure that uncertainty quantification remains accurate for both tasks. Post hoc isotonic regression is applied if miscalibration is detected.

### 3.14. Clinical Translation and Validation

Clinical translation of generative models requires rigorous validation to ensure that synthesized images maintain anatomical fidelity and do not introduce artifacts that could mislead diagnostic algorithms [101]. Studies comparing diffusion-based image synthesis with traditional augmentation approaches have shown that synthetic images exhibit anatomical fidelity and diversity while helping models learn representations consistent with human expert opinions [102]. In contrast, traditional augmented images may impede model generalizability, highlighting the importance of sophisticated generative approaches.

Quantitative evaluation frameworks have been developed to assess the quality of synthesized medical images across multiple dimensions. Two radiologists rating synthetic images regarding realistic appearance, anatomical correctness, and slice consistency have provided valuable insights into the clinical utility of diffusion-generated medical data [103]. Studies using magnetic resonance imaging and computed tomography datasets have demonstrated that diffusion models can synthesize high-quality medical data suitable for self-supervised pre training and performance improvement in downstream tasks [104].

Memorization analysis has revealed important considerations for the clinical deployment of generative models in medical imaging. Comparative studies between diffusion models and generative adversarial networks have shown that diffusion models are more likely to memorize training images, particularly for small datasets and when using 2D slices from 3D volumes [105]. These findings emphasize the importance of careful evaluation when using generative models for data-sharing applications, requiring researchers to quantify memorization and data leakage to ensure patient privacy protection. Proper validation protocols are essential for establishing the safety and reliability of synthetic medical images in clinical practice [106].

## 4. Discussion

The integration of Bayesian graphical models with advanced medical imaging techniques has created unprecedented opportunities for improving the diagnosis, monitoring, and treatment of joint degeneration [107]. The comprehensive framework presented in this review demonstrates how probabilistic modeling approaches can address the inherent uncertainty in medical imaging while providing clinically actionable insights across multiple scales of biological organization [108]. From molecular biomarkers to tissue-level structural changes, Bayesian methods enable the synthesis of heterogeneous data sources into unified analytical frameworks that support evidence-based clinical decision-making. This review directly addressed the central question of whether integrating Bayesian graphical models with multiscale medical imaging improves detection, analysis, and prediction of joint degeneration compared to traditional methods. The synthesis of evidence across Bayesian inference, multiscale radiomics, GNN-based modeling, spatiotemporal tracking, and multimodal integration supports the conclusion that such integrative approaches provide a more comprehensive and probabilistically robust characterization of joint disease [107]. Conclusions align with the presented evidence, while acknowledging limitations such as computational demands, data requirements, and interpretability challenges [108].

### 4.1. Clinical Implementation Challenges

The translation of sophisticated Bayesian models into routine clinical practice faces several significant challenges that must be addressed to realize the full potential of these approaches [109]. Technical standardization of both biomarker measurement and imaging acquisition protocols is essential for ensuring reproducibility across different healthcare environments and imaging systems. Clinical validation studies must demonstrate incremental value over existing diagnostic methods while proving meaningful improvements in patient outcomes and management decisions [110]. The additional complexity and computational requirements associated with Bayesian models necessitate careful consideration of cost-effectiveness, workflow integration, and training requirements for healthcare providers.

Implementation science frameworks such as the Consolidated Framework for Implementation Research provide structured approaches for addressing these challenges through comprehensive needs assessments, stakeholder engagement, and systematic workflow analysis [111]. Physician training programs must encompass theoretical knowledge of underlying scientific principles, procedural skills for equipment operation and quality assessment, interpretive capabilities for pattern recognition and artifact identification, and professional attitudes regarding appropriate utilization and ongoing learning [112]. The effectiveness of training programs can be assessed through knowledge tests, procedural checklists, interpretation metrics, and workplace observation studies.

Regulatory pathways for novel diagnostic approaches require careful navigation of approval processes while ensuring patient safety and clinical efficacy. The Food and Drug Administration’s 510 (k) clearance process for Class II imaging devices and European Union Medical Device Regulation requirements mandate comprehensive analytical and clinical validation studies [113]. Emerging frameworks for artificial intelligence and machine learning applications address algorithm transparency, continuous learning capabilities, and post-market surveillance requirements [114]. Early regulatory consultation, appropriate predicate device selection, and robust validation documentation are essential for successful translation of diagnostic innovations into clinical tools.

### 4.2. Potential Solutions for Widespread Implementation

Several promising research directions are emerging that will likely shape the future development of Bayesian graphical models for joint degeneration analysis. High-throughput biomarker discovery using next-generation sequencing, mass spectrometry-based proteomics, and nuclear magnetic resonance metabolomics will continue to expand the molecular landscape available for integration with imaging data [89]. Single-cell analytics including RNA sequencing, ATAC-seq, proteomics, and spatial transcriptomics provide unprecedented resolution for characterizing cellular heterogeneity in joint tissues. These technological advances will enable more sophisticated models that capture disease mechanisms across multiple biological scales [50].

Advanced imaging technologies including ultra-high-field MRI at 7 Tesla and beyond offer improved resolution and novel contrast mechanisms for detecting early cartilage and synovial changes [86]. Molecular imaging techniques such as positron emission tomography and single-photon emission computed tomography enable visualization of specific biological processes at molecular and cellular levels. Hybrid imaging systems that combine molecular and anatomical information provide enhanced diagnostic precision while functional imaging approaches capture dynamic physiological processes [93]. Although these technologies remain primarily research tools, they promise earlier detection, improved patient stratification, and enhanced therapeutic monitoring.

Explainable artificial intelligence techniques will become increasingly important for clinical adoption of complex Bayesian models. Methods ranging from interpretable models like decision trees and attention-based networks to post hoc techniques such as SHAP, LIME, and Grad-CAM will provide explicit model evaluations that enable clinical understanding and trust [81]. Federated learning approaches will enhance model robustness while preserving patient privacy through collaborative training across institutions without exposing raw patient data [74]. These developments will enable the creation of more generalizable models that reflect global diversity in demographics, imaging modalities, and disease presentations.

### 4.3. Bayesion Graphical Models and Multimodal Imaging: Advancing Precision Medicine

The convergence of Bayesian graphical models with advanced medical imaging technologies represents a paradigm shift toward precision medicine approaches in joint degeneration analysis [66]. Multimodal integration strategies that combine structural MRI, functional imaging, biochemical biomarkers, and clinical data provide comprehensive characterizations of disease state and progression risk. These integrated approaches enable the identification of disease subtypes with distinct molecular mechanisms, structural patterns, and therapeutic responses, supporting the development of personalized treatment strategies.

Point-of-care applications utilizing portable imaging technologies, rapid biomarker assays, and mobile health platforms have the potential to extend advanced diagnostic capabilities beyond specialized centers to primary care and community settings [56]. Telemedicine integration can provide remote expert consultation and monitoring, particularly valuable for underserved populations and resource-limited settings. These developments align directly with the special issue focus on addressing healthcare disparities and improving diagnostic performance in developing regions where access to experienced specialists may be limited.

The systematic application of Bayesian graphical models to joint degeneration analysis offers transformative potential for improving patient outcomes through earlier detection, more accurate risk stratification, and personalized therapeutic targeting [34]. By providing probabilistic assessments of disease progression and treatment response, these approaches enable clinicians to make evidence-based decisions that optimize individual patient care while advancing our understanding of joint pathophysiology. The continued development and validation of these methods will be essential for addressing the growing global burden of joint degeneration and musculoskeletal disorders.

## 5. Conclusions

The collective findings from this review highlight the unique advantages of Bayesian graphical models when applied alongside multiscale medical imaging. By enabling the integration of diverse data streams—ranging from molecular biomarkers to high-resolution anatomical scans—these approaches provide a unified framework capable of capturing the complexity of joint degeneration. This synthesis not only facilitates earlier and more accurate detection of disease but also supports a richer understanding of underlying biological mechanisms that drive progression and therapeutic response.

Beyond their diagnostic utility, Bayesian methods offer a principled means of quantifying uncertainty, a feature that is critical in clinical decision-making where incomplete or noisy data are common. The probabilistic nature of these models allows for the continuous refinement of predictions as new evidence becomes available, fostering adaptive and personalized care pathways. Their capacity to incorporate temporal, spatial, and connectivity information across biological scales positions them as a powerful tool for identifying patient subgroups, tailoring interventions, and monitoring outcomes with greater precision.

Looking ahead, the integration of Bayesian modeling with next-generation imaging technologies, explainable artificial intelligence, and federated learning frameworks promises to further enhance their clinical impact. Achieving this potential will require rigorous validation in diverse populations, alignment with regulatory standards, and thoughtful integration into clinical workflows. If these challenges are met, Bayesian graphical models could play a central role in transforming joint degeneration management, advancing precision medicine, and addressing the growing global burden of musculoskeletal disease.

## Data Availability

No new data were created or analyzed in this study.
